# *trans*-Iodido(pyrazinyl-κ*C* ^2^)bis­(triphenyl­phosphane-κ*P*)palladium(II)

**DOI:** 10.1107/S1600536812043589

**Published:** 2012-10-31

**Authors:** Yao-Jen Tu, Hsiao-Fen Wang, Gene-Hsiang Lee, Kuang-Hway Yih, Xiao-Yan Tang

**Affiliations:** a College of Environmental Sciences and Engineering, Peking University, Beijing 100871, People’s Republic of China; b Department of Hair Styling and Design, Hungkuang University, Shalu 433, Taichung, Taiwan, ROC; c Instrumentation Center, College of Science, National Taiwan University, Taipei 106, Taiwan, ROC; d Department of Applied Cosmetology, Hungkuang University, Shalu 433, Taichung, Taiwan, ROC

## Abstract

There are two independent mol­ecules with similar configurations in the asymmetric unit of the title complex, [Pd(C_4_H_3_N_2_)I(C_18_H_15_P)_2_]. In each mol­ecule, the geometry around the Pd atom is distorted square-planar, with the Pd atom displaced by 0.0549 (12) and 0.0734 (13) Å from the least-squares plane of the I—P—P—C atoms. The PPh_3_ ligands are in *trans* positions, with P—Pd—P angles of 173.12 (4) and 170.29 (4)°, while the pyrazinyl ligands and I atoms, also *trans* to each other, form C—Pd—I angles of 179.38 (12) and 178.44 (12)°. In the crystal, C—H⋯π inter­actions occur, resulting in a three-dimensional supramolecular architecture.

## Related literature

For reactions in organic synthesis that form C—C bonds, see: Steffen *et al.* (2005 ▶); Beeby *et al.* (2004 ▶); Chin *et al.* (1988 ▶); Dobrzynski & Angelici (1975 ▶). For Pd—C(carbene) bond lengths, see: Cardin *et al.* (1972 ▶) and for Pd—I bond lengths, see: Yih *et al.* (2009 ▶). For intra­molecular π–π inter­actions, see: Bustos *et al.* (2006 ▶). For a Pd–pyrimidinyl complex, see: Wang *et al.* (2011 ▶).
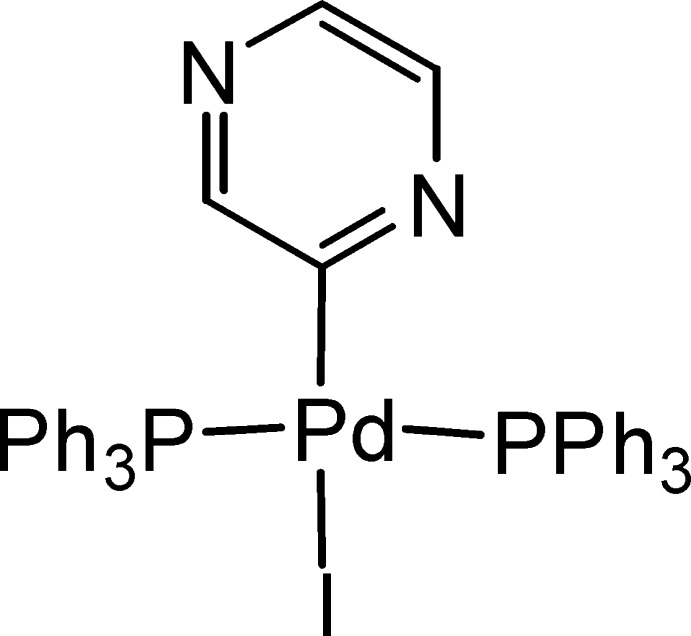


## Experimental

### 

#### Crystal data

[Pd(C_4_H_3_N_2_)I(C_18_H_15_P)_2_]
*M* *_r_* = 836.92
Monoclinic, 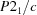

*a* = 21.5786 (9) Å
*b* = 19.8596 (9) Å
*c* = 16.9192 (8) Å
β = 106.6952 (11)°
*V* = 6945.0 (5) Å^3^
*Z* = 8
Mo *K*α radiation
μ = 1.55 mm^−1^
*T* = 150 K
0.27 × 0.20 × 0.16 mm

#### Data collection

Bruker SMART APEX CCD area-detector diffractometer
Absorption correction: multi-scan (*SADABS*; Bruker, 2001 ▶) *T*_min_ = 0.680, *T*_max_ = 0.790
40984 measured reflections
15924 independent reflections
11520 reflections with *I* > 2σ(*I*)
*R*_int_ = 0.055

#### Refinement

*R*[*F* ^2^ > 2σ(*F* ^2^)] = 0.048
*wR*(*F* ^2^) = 0.101
*S* = 1.04
15924 reflections
829 parameters
H-atom parameters constrained
Δρ_max_ = 1.35 e Å^−3^
Δρ_min_ = −0.55 e Å^−3^

### 

Data collection: *SMART* (Bruker, 2007 ▶); cell refinement: *SAINT* (Bruker, 2007 ▶); data reduction: *SAINT*; program(s) used to solve structure: *SHELXS97* (Sheldrick, 2008 ▶); program(s) used to refine structure: *SHELXL97* (Sheldrick, 2008 ▶); molecular graphics: *XP* in *SHELXTL* (Sheldrick, 2008 ▶); software used to prepare material for publication: *SHELXTL*.

## Supplementary Material

Crystal structure: contains datablock(s) I, global. DOI: 10.1107/S1600536812043589/bg2480sup1.cif

Structure factors: contains datablock(s) I. DOI: 10.1107/S1600536812043589/bg2480Isup2.hkl

Additional supplementary materials:  crystallographic information; 3D view; checkCIF report

## Figures and Tables

**Table 1:** Hydrogen-bond geometry (Å, °) *Cg*1, *Cg*2, *Cg*3, *Cg*4 and *Cg*5 are the centroids of the C11–C16, C17–C22, C51–C56, C63–C68 and C75–C80 rings, respectively.

*D*—H⋯*A*	*D*—H	H⋯*A*	*D*⋯*A*	*D*—H⋯*A*
C2—H2⋯*Cg*5^i^	0.95	2.99	3.898 (5)	160
C7—H7⋯*Cg*3	0.95	3.00	3.930 (5)	168
C25—H25⋯*Cg*1^ii^	0.95	2.91	3.769 (5)	151
C42—H42⋯*Cg*2^ii^	0.95	3.00	3.944 (5)	176
C53—H53⋯*Cg*4^i^	0.95	2.80	3.617 (5)	145

Symmetry codes: (i) 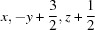; (ii) 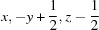.

**Fig. 1.**
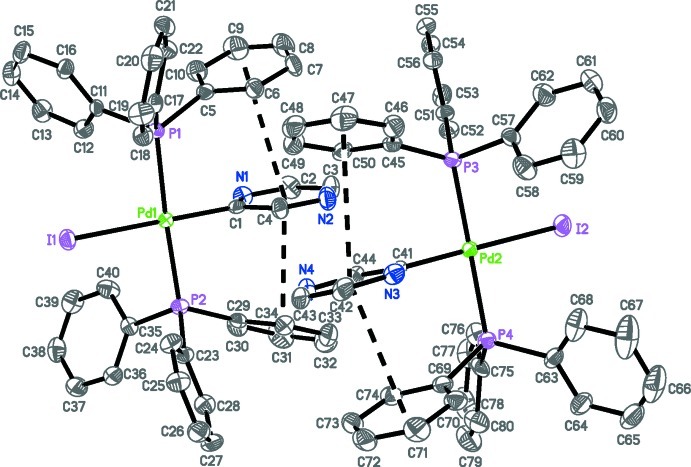
The molecular structure of (I), showing the atom-numbering scheme and the 50% probability displacement ellipsoids. Dashed lines represent the π-π interactions.

**Fig. 2.**
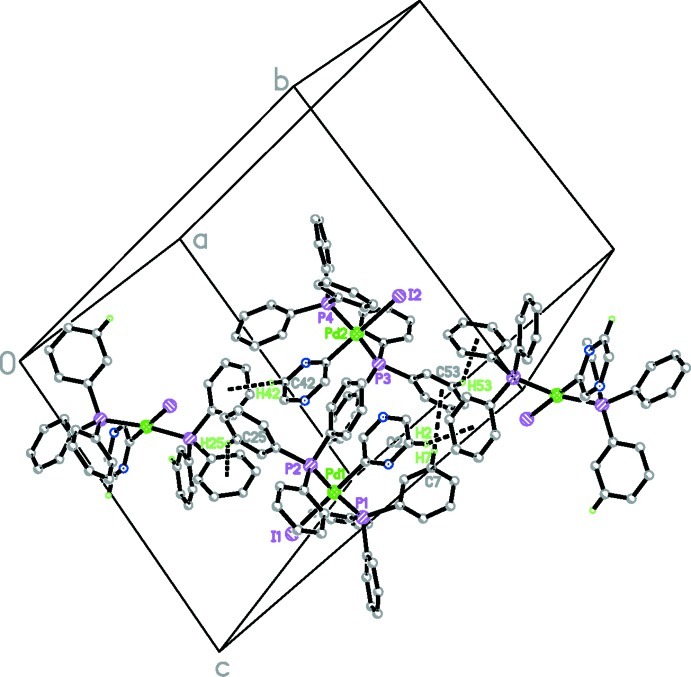
The molecular structure of (I), showing the intermolecular C—H···π hydrogen bond interactions.

## supplementary crystallographic information

### Comment

Processes leading to the formation of C—C bonds and cathalized by Palladium complexes are among the most important reactions in organic synthesis (Dobrzynski & Angelici, 1975). Intramolecular reductive elimination of the Pd—N binuclear complex [Pd(µ-C_9_H_6_N)(µ-dppm)]_2_(Cl)_2_ yielding the organic compound 2,2'-biquinoline has been reported (Chin *et al.*, 1988). A pyridyl-bridged palladium complex was reported as an effective precatalyst for the Suzuki cross-coupling reactions of a variety organoboronic acids and aryl bromides (Beeby, *et al.*, 2004). Pyrazinyl nickel complexes have been used to as a catalyst for C—C coupling reactions (Steffen *et al.*, 2005), but to our knowledge, no pyrazinyl palladium crystal structure has been described.

For the synthesis of the pyrazinyl title compound, complex [Pd(PPh_3_)_4_] was used to react with 2-iodopyrazine in dichloromethane at room temperature. As a result, a two triphenylphosphine displaced complex [Pd(I)(C_4_H_3_N_2_)(PPh_3_)_2_] was isolated with 98% yield. The X-ray crystal structure analysis has been carried out to provide structural parameters.

The molecular structure (with two independent molecules) is shown in Fig. 1. There are small difference in bond distances (in the range of 0.001–0.027 Å) and bond angles (in the range of 0.05–0.7.46°) between them, mainly around the metal atoms. The palladium atom has a distorted square planar geometry, while being displaced by 0.0549 (12) Å (0.0734 (13) Å) from the least-squares plane Angles around the Pd center lay within ± 2.79° of 90°. The average Pd—C1(C41) bond distance, 1.998 (4) Å, is longer than reported Pd^II^-carbon(carbonyl) distances, and similar to those of Pd—C(carbene) distances (Cardin *et al.*, 1972). The PPh_3_ ligands are in *trans* position: P—Pd—P(av), 171.71 (4)°, while the pyrazinyl ligand and iodine atom, also *trans* to each other, present a C—Pd—I(av) of 178.91 (12)°. The phosphorus atoms approach tetrahedral geometry as expected, with a maximun deviation from idealized tetrahedral geometry for C5—P1—Pd = 119.13 (14)°. The average Pd—N1(N(3) bond distance of 2.899 (4) Å indicates no bonding interaction between the nitrogen atom and palladium metal atom. Within the pyrazinyl ligand itself, the geometry is consistent with a significant partial double bond character in the C—C and C—N bond. The C—N(av) bond distances (1.321 (5), 1.353 (6) Å) are typical for a C—N bond having partial double bond character and are certainly much shorter than a normal C—N (1.47 Å) single bond. The average Pd—C (1.998 Å), Pd—P (2.3375, 2.3255 Å) and Pd—I (2.7007 Å) coordination lengths of (I) are in agreement with reported values (Yih *et al.*, 2009).

There are five C—H···π intermolecular hydrogen bond interactions (Fig. 2 and Table 1) and intramolecular π-π interactions (Fig. 1). The pyrazinyl ligand (N1, N2, C1 > C4) and two phenyl rings (C5 > C10 and C29 > C34) from the *trans* triphenylphosphanes respectively are nearly parallel, with intercentroid distances of 3.564 (3) and 3.677 (3) Å, and a shortest inter-ring distance of 3.120 (2) and 3,317 (2) Å. A similar effect is observed in the remaining molecule, where the intercentroid distances between the pyrazinyl ligand (N3, N4, C41 > C44) and the phenyl rings (C45 > C50 and C69 > C74) are 3.716 (3) and 3.446 (3) Å, with a shortest inter-ring distance of 3.225 (2) and 3,119 (2) Å.

### Experimental

The synthesis of the title compound (I) was carried out as follows. CH_2_Cl_2_ (20 ml) was added to a flask (100 ml) containing Pd(PPh_3_)_4_ (1.155 g, 1.0 mmol) and 2-iodiopyrazine (0.248 g, 1.2 mmol) at ambient temperature. The mixture was stirred for about 10 min. The solvent was concentrated to 10 ml, and 20 ml of diethyl ether was added to the solution. The pale-yellow solids were formed which were isolated by filtration (G4), washed with n-hexane (2 *x* 10 ml) and subsequently dried under vacuum yielding 0.82 g (98%) of [Pd(PPh_3_)_2_(C_4_H_3_N_2_)I], (I).

### Refinement

H atoms were positioned geometrically and refined using a riding model, with C—H = 0.95 Å and with *U*_iso_(H) = 1.2 times *U*_eq_(C).

### Crystal data

**Table:**

[Pd(C_4_H_3_N_2_)I(C_18_H_15_P)_2_]	*F*(000) = 3328
*M**_r_* = 836.92	*D*_x_ = 1.601 Mg m^−^^3^
Monoclinic, *P*2_1_/*c*	Mo *K*α radiation, λ = 0.71073 Å
Hall symbol: -P 2ybc	Cell parameters from 4895 reflections
*a* = 21.5786 (9) Å	θ = 2.2–25.3°
*b* = 19.8596 (9) Å	µ = 1.55 mm^−^^1^
*c* = 16.9192 (8) Å	*T* = 150 K
β = 106.6952 (11)°	Block, colorless
*V* = 6945.0 (5) Å^3^	0.27 × 0.20 × 0.16 mm
*Z* = 8	

### Data collection

**Table:**

Bruker SMART APEX CCD area-detector diffractometer	15924 independent reflections
Radiation source: fine-focus sealed tube	11520 reflections with *I* > 2σ(*I*)
Graphite monochromator	*R*_int_ = 0.055
ω scans	θ_max_ = 27.5°, θ_min_ = 1.0°
Absorption correction: multi-scan (*SADABS*; Bruker, 2001)	*h* = −28→18
*T*_min_ = 0.680, *T*_max_ = 0.790	*k* = −25→22
40984 measured reflections	*l* = −21→21

### Refinement

**Table:**

Refinement on *F*^2^	Primary atom site location: structure-invariant direct methods
Least-squares matrix: full	Secondary atom site location: difference Fourier map
*R*[*F*^2^ > 2σ(*F*^2^)] = 0.048	Hydrogen site location: inferred from neighbouring sites
*wR*(*F*^2^) = 0.101	H-atom parameters constrained
*S* = 1.04	*w* = 1/[σ^2^(*F*_o_^2^) + (0.033*P*)^2^ + 0.398*P*] where *P* = (*F*_o_^2^ + 2*F*_c_^2^)/3
15924 reflections	(Δ/σ)_max_ = 0.003
829 parameters	Δρ_max_ = 1.35 e Å^−^^3^
0 restraints	Δρ_min_ = −0.55 e Å^−^^3^

### Special details

**Table:**

Geometry. All e.s.d.'s (except the e.s.d. in the dihedral angle between two l.s. planes) are estimated using the full covariance matrix. The cell e.s.d.'s are taken into account individually in the estimation of e.s.d.'s in distances, angles and torsion angles; correlations between e.s.d.'s in cell parameters are only used when they are defined by crystal symmetry. An approximate (isotropic) treatment of cell e.s.d.'s is used for estimating e.s.d.'s involving l.s. planes.
Refinement. Refinement of *F*^2^ against ALL reflections. The weighted *R*-factor *wR* and goodness of fit *S* are based on *F*^2^, conventional *R*-factors *R* are based on *F*, with *F* set to zero for negative *F*^2^. The threshold expression of *F*^2^ > σ(*F*^2^) is used only for calculating *R*-factors(gt) *etc*. and is not relevant to the choice of reflections for refinement. *R*-factors based on *F*^2^ are statistically about twice as large as those based on *F*, and *R*- factors based on ALL data will be even larger.

### Fractional atomic coordinates and isotropic or equivalent isotropic displacement parameters (Å^2^)

**Table:**

	*x*	*y*	*z*	*U*_iso_*/*U*_eq_	
Pd1	0.146035 (15)	0.406204 (16)	0.895828 (18)	0.01645 (8)	
I1	0.089411 (14)	0.285212 (15)	0.898708 (18)	0.02538 (8)	
P1	0.23255 (5)	0.37985 (6)	1.01093 (6)	0.0168 (2)	
P2	0.05913 (5)	0.44510 (6)	0.78820 (7)	0.0195 (2)	
N1	0.17522 (18)	0.54643 (19)	0.9394 (2)	0.0253 (9)	
N2	0.25835 (19)	0.5641 (2)	0.8410 (2)	0.0319 (10)	
C1	0.18713 (19)	0.4964 (2)	0.8938 (2)	0.0193 (9)	
C2	0.2052 (2)	0.6052 (2)	0.9359 (3)	0.0298 (11)	
H2	0.1979	0.6421	0.9680	0.036*	
C3	0.2459 (2)	0.6137 (2)	0.8876 (3)	0.0339 (12)	
H3	0.2659	0.6562	0.8872	0.041*	
C4	0.2281 (2)	0.5069 (2)	0.8446 (3)	0.0242 (10)	
H4	0.2347	0.4706	0.8114	0.029*	
C5	0.2836 (2)	0.4508 (2)	1.0597 (3)	0.0214 (10)	
C6	0.3266 (2)	0.4770 (2)	1.0200 (3)	0.0255 (10)	
H6	0.3328	0.4549	0.9731	0.031*	
C7	0.3607 (2)	0.5359 (2)	1.0495 (3)	0.0357 (13)	
H7	0.3898	0.5539	1.0223	0.043*	
C8	0.3524 (3)	0.5679 (3)	1.1180 (3)	0.0419 (14)	
H8	0.3754	0.6082	1.1377	0.050*	
C9	0.3109 (3)	0.5415 (3)	1.1574 (3)	0.0415 (14)	
H9	0.3058	0.5633	1.2051	0.050*	
C10	0.2760 (2)	0.4833 (2)	1.1288 (3)	0.0314 (11)	
H10	0.2470	0.4658	1.1565	0.038*	
C11	0.2050 (2)	0.3462 (2)	1.0950 (2)	0.0181 (9)	
C12	0.1520 (2)	0.3765 (2)	1.1116 (3)	0.0278 (11)	
H12	0.1295	0.4117	1.0770	0.033*	
C13	0.1320 (2)	0.3558 (2)	1.1782 (3)	0.0309 (11)	
H13	0.0967	0.3779	1.1901	0.037*	
C14	0.1626 (2)	0.3036 (2)	1.2273 (3)	0.0304 (12)	
H14	0.1484	0.2897	1.2729	0.036*	
C15	0.2141 (2)	0.2713 (2)	1.2104 (3)	0.0310 (12)	
H15	0.2344	0.2343	1.2433	0.037*	
C16	0.2363 (2)	0.2931 (2)	1.1448 (3)	0.0236 (10)	
H16	0.2725	0.2718	1.1341	0.028*	
C17	0.2913 (2)	0.3187 (2)	0.9962 (2)	0.0197 (9)	
C18	0.2746 (2)	0.2741 (2)	0.9303 (3)	0.0252 (10)	
H18	0.2327	0.2758	0.8921	0.030*	
C19	0.3193 (2)	0.2269 (2)	0.9201 (3)	0.0320 (12)	
H19	0.3078	0.1966	0.8748	0.038*	
C20	0.3802 (2)	0.2238 (2)	0.9754 (3)	0.0321 (12)	
H20	0.4104	0.1914	0.9680	0.038*	
C21	0.3972 (2)	0.2672 (2)	1.0407 (3)	0.0285 (11)	
H21	0.4392	0.2650	1.0787	0.034*	
C22	0.3530 (2)	0.3145 (2)	1.0515 (3)	0.0261 (10)	
H22	0.3650	0.3444	1.0972	0.031*	
C23	0.0450 (2)	0.4064 (2)	0.6870 (2)	0.0206 (9)	
C24	0.0775 (2)	0.3482 (2)	0.6767 (3)	0.0274 (11)	
H24	0.1054	0.3262	0.7233	0.033*	
C25	0.0692 (2)	0.3222 (2)	0.5983 (3)	0.0279 (11)	
H25	0.0918	0.2825	0.5918	0.034*	
C26	0.0291 (2)	0.3527 (2)	0.5299 (3)	0.0309 (11)	
H26	0.0238	0.3341	0.4766	0.037*	
C27	−0.0038 (2)	0.4113 (3)	0.5392 (3)	0.0311 (12)	
H27	−0.0317	0.4329	0.4922	0.037*	
C28	0.0044 (2)	0.4376 (2)	0.6168 (3)	0.0262 (10)	
H28	−0.0179	0.4776	0.6229	0.031*	
C29	0.0602 (2)	0.5342 (2)	0.7604 (3)	0.0228 (10)	
C30	0.0258 (2)	0.5822 (2)	0.7892 (3)	0.0315 (12)	
H30	0.0006	0.5693	0.8245	0.038*	
C31	0.0279 (2)	0.6497 (3)	0.7667 (4)	0.0439 (14)	
H31	0.0039	0.6828	0.7859	0.053*	
C32	0.0657 (3)	0.6677 (3)	0.7156 (4)	0.0460 (15)	
H32	0.0668	0.7133	0.6991	0.055*	
C33	0.1010 (2)	0.6209 (3)	0.6892 (3)	0.0378 (13)	
H33	0.1277	0.6340	0.6559	0.045*	
C34	0.0982 (2)	0.5543 (2)	0.7106 (3)	0.0310 (11)	
H34	0.1225	0.5217	0.6912	0.037*	
C35	−0.0150 (2)	0.4370 (2)	0.8193 (3)	0.0212 (10)	
C36	−0.0760 (2)	0.4286 (2)	0.7642 (3)	0.0320 (12)	
H36	−0.0808	0.4256	0.7067	0.038*	
C37	−0.1299 (2)	0.4245 (3)	0.7925 (3)	0.0398 (14)	
H37	−0.1714	0.4186	0.7543	0.048*	
C38	−0.1241 (2)	0.4289 (2)	0.8757 (3)	0.0339 (12)	
H38	−0.1613	0.4258	0.8948	0.041*	
C39	−0.0640 (2)	0.4378 (2)	0.9306 (3)	0.0340 (12)	
H39	−0.0598	0.4419	0.9879	0.041*	
C40	−0.0094 (2)	0.4408 (2)	0.9030 (3)	0.0291 (11)	
H40	0.0321	0.4456	0.9417	0.035*	
Pd2	0.348046 (15)	0.592426 (16)	0.622033 (18)	0.01642 (8)	
I2	0.406173 (15)	0.713311 (15)	0.626859 (18)	0.02865 (8)	
P3	0.42975 (5)	0.55229 (6)	0.73396 (7)	0.0190 (2)	
P4	0.27144 (6)	0.61773 (6)	0.49785 (7)	0.0207 (3)	
N3	0.32016 (18)	0.45176 (18)	0.5822 (2)	0.0247 (9)	
N4	0.21891 (19)	0.4440 (2)	0.6553 (2)	0.0313 (10)	
C41	0.3031 (2)	0.5044 (2)	0.6191 (2)	0.0176 (9)	
C42	0.2857 (2)	0.3946 (2)	0.5807 (3)	0.0306 (11)	
H42	0.2967	0.3555	0.5553	0.037*	
C43	0.2350 (2)	0.3918 (2)	0.6152 (3)	0.0334 (12)	
H43	0.2107	0.3513	0.6104	0.040*	
C44	0.2532 (2)	0.4990 (2)	0.6557 (3)	0.0240 (10)	
H44	0.2432	0.5376	0.6829	0.029*	
C45	0.4259 (2)	0.4636 (2)	0.7618 (2)	0.0206 (9)	
C46	0.4659 (2)	0.4140 (2)	0.7458 (3)	0.0302 (11)	
H46	0.4995	0.4258	0.7224	0.036*	
C47	0.4572 (2)	0.3475 (2)	0.7640 (3)	0.0368 (12)	
H47	0.4844	0.3137	0.7522	0.044*	
C48	0.4096 (2)	0.3298 (2)	0.7987 (3)	0.0339 (12)	
H48	0.4039	0.2840	0.8111	0.041*	
C49	0.3701 (3)	0.3784 (3)	0.8156 (3)	0.0344 (12)	
H49	0.3369	0.3661	0.8396	0.041*	
C50	0.3784 (2)	0.4451 (2)	0.7979 (2)	0.0249 (10)	
H50	0.3513	0.4786	0.8106	0.030*	
C51	0.4455 (2)	0.5931 (2)	0.8343 (2)	0.0190 (9)	
C52	0.4094 (2)	0.6479 (2)	0.8460 (3)	0.0252 (10)	
H52	0.3764	0.6657	0.8009	0.030*	
C53	0.4213 (2)	0.6768 (2)	0.9234 (3)	0.0278 (11)	
H53	0.3966	0.7146	0.9311	0.033*	
C54	0.4686 (2)	0.6513 (2)	0.9892 (3)	0.0260 (10)	
H54	0.4764	0.6713	1.0421	0.031*	
C55	0.5049 (2)	0.5964 (2)	0.9783 (3)	0.0273 (11)	
H55	0.5378	0.5789	1.0237	0.033*	
C56	0.4931 (2)	0.5671 (2)	0.9016 (3)	0.0240 (10)	
H56	0.5176	0.5291	0.8944	0.029*	
C57	0.5029 (2)	0.5600 (2)	0.7017 (3)	0.0220 (10)	
C58	0.5026 (2)	0.5306 (2)	0.6273 (3)	0.0334 (12)	
H58	0.4662	0.5053	0.5968	0.040*	
C59	0.5559 (2)	0.5383 (2)	0.5973 (3)	0.0369 (13)	
H59	0.5557	0.5178	0.5465	0.044*	
C60	0.6084 (2)	0.5750 (3)	0.6400 (3)	0.0349 (12)	
H60	0.6446	0.5801	0.6193	0.042*	
C61	0.6082 (2)	0.6047 (3)	0.7135 (3)	0.0402 (13)	
H61	0.6445	0.6305	0.7433	0.048*	
C62	0.5558 (2)	0.5974 (3)	0.7447 (3)	0.0362 (12)	
H62	0.5563	0.6180	0.7955	0.043*	
C63	0.3096 (2)	0.6476 (2)	0.4213 (3)	0.0266 (11)	
C64	0.2779 (3)	0.6866 (3)	0.3541 (3)	0.0487 (16)	
H64	0.2354	0.7027	0.3480	0.058*	
C65	0.3104 (4)	0.7020 (3)	0.2948 (3)	0.062 (2)	
H65	0.2892	0.7283	0.2480	0.074*	
C66	0.3709 (4)	0.6800 (3)	0.3034 (4)	0.065 (2)	
H66	0.3918	0.6911	0.2627	0.077*	
C67	0.4028 (4)	0.6415 (3)	0.3702 (4)	0.062 (2)	
H67	0.4456	0.6261	0.3762	0.074*	
C68	0.3716 (3)	0.6258 (3)	0.4285 (3)	0.0420 (14)	
H68	0.3934	0.5991	0.4748	0.050*	
C69	0.2243 (2)	0.5458 (2)	0.4453 (3)	0.0244 (10)	
C70	0.2445 (2)	0.5079 (2)	0.3884 (3)	0.0293 (11)	
H70	0.2804	0.5223	0.3709	0.035*	
C71	0.2121 (3)	0.4490 (2)	0.3572 (3)	0.0362 (12)	
H71	0.2249	0.4242	0.3165	0.043*	
C72	0.1617 (2)	0.4261 (3)	0.3847 (3)	0.0363 (13)	
H72	0.1411	0.3848	0.3644	0.044*	
C73	0.1411 (2)	0.4625 (2)	0.4409 (3)	0.0313 (11)	
H73	0.1058	0.4469	0.4591	0.038*	
C74	0.1723 (2)	0.5227 (2)	0.4716 (3)	0.0270 (11)	
H74	0.1581	0.5482	0.5106	0.032*	
C75	0.2096 (2)	0.6772 (2)	0.5041 (3)	0.0263 (11)	
C76	0.2169 (2)	0.7142 (2)	0.5748 (3)	0.0320 (11)	
H76	0.2556	0.7101	0.6188	0.038*	
C77	0.1684 (3)	0.7577 (3)	0.5833 (4)	0.0449 (14)	
H77	0.1743	0.7831	0.6325	0.054*	
C78	0.1129 (3)	0.7634 (3)	0.5207 (4)	0.0504 (17)	
H78	0.0794	0.7921	0.5272	0.061*	
C79	0.1040 (3)	0.7285 (3)	0.4484 (4)	0.0503 (16)	
H79	0.0653	0.7340	0.4047	0.060*	
C80	0.1521 (2)	0.6850 (2)	0.4392 (3)	0.0405 (13)	
H80	0.1462	0.6606	0.3893	0.049*	

### Atomic displacement parameters (Å^2^)

**Table:**

	*U*^11^	*U*^22^	*U*^33^	*U*^12^	*U*^13^	*U*^23^
Pd1	0.01639 (17)	0.01662 (18)	0.01584 (16)	−0.00067 (13)	0.00386 (13)	0.00095 (13)
I1	0.02899 (17)	0.02145 (17)	0.02611 (16)	−0.00769 (13)	0.00859 (13)	−0.00107 (12)
P1	0.0177 (6)	0.0164 (6)	0.0164 (5)	−0.0003 (5)	0.0049 (4)	−0.0006 (4)
P2	0.0185 (6)	0.0211 (6)	0.0179 (5)	0.0031 (5)	0.0037 (5)	0.0004 (5)
N1	0.026 (2)	0.025 (2)	0.027 (2)	0.0014 (17)	0.0101 (17)	−0.0026 (17)
N2	0.032 (2)	0.034 (3)	0.033 (2)	−0.010 (2)	0.0145 (19)	0.0015 (19)
C1	0.014 (2)	0.024 (3)	0.018 (2)	0.0027 (18)	0.0016 (17)	0.0060 (18)
C2	0.030 (3)	0.019 (3)	0.042 (3)	0.005 (2)	0.012 (2)	−0.008 (2)
C3	0.041 (3)	0.020 (3)	0.043 (3)	−0.005 (2)	0.016 (3)	0.004 (2)
C4	0.025 (2)	0.025 (3)	0.026 (2)	−0.003 (2)	0.013 (2)	−0.0016 (19)
C5	0.022 (2)	0.017 (2)	0.022 (2)	0.0007 (19)	0.0012 (19)	0.0017 (18)
C6	0.024 (3)	0.020 (3)	0.032 (3)	0.002 (2)	0.007 (2)	0.003 (2)
C7	0.021 (3)	0.029 (3)	0.055 (3)	−0.007 (2)	0.008 (2)	0.011 (3)
C8	0.040 (3)	0.024 (3)	0.054 (4)	−0.009 (2)	0.002 (3)	−0.003 (3)
C9	0.049 (4)	0.027 (3)	0.043 (3)	−0.004 (3)	0.005 (3)	−0.011 (2)
C10	0.035 (3)	0.024 (3)	0.033 (3)	−0.003 (2)	0.006 (2)	−0.003 (2)
C11	0.020 (2)	0.020 (2)	0.015 (2)	−0.0062 (18)	0.0066 (17)	−0.0005 (17)
C12	0.026 (3)	0.031 (3)	0.026 (2)	0.001 (2)	0.007 (2)	0.004 (2)
C13	0.033 (3)	0.038 (3)	0.028 (3)	−0.003 (2)	0.017 (2)	−0.003 (2)
C14	0.046 (3)	0.028 (3)	0.022 (2)	−0.013 (2)	0.017 (2)	−0.007 (2)
C15	0.048 (3)	0.020 (3)	0.022 (2)	−0.009 (2)	0.007 (2)	0.0019 (19)
C16	0.031 (3)	0.021 (3)	0.020 (2)	−0.004 (2)	0.0088 (19)	−0.0005 (18)
C17	0.020 (2)	0.021 (2)	0.021 (2)	−0.0027 (19)	0.0111 (18)	0.0019 (18)
C18	0.025 (3)	0.025 (3)	0.026 (2)	0.000 (2)	0.008 (2)	−0.0002 (19)
C19	0.036 (3)	0.031 (3)	0.029 (3)	0.005 (2)	0.010 (2)	−0.006 (2)
C20	0.034 (3)	0.022 (3)	0.044 (3)	0.010 (2)	0.019 (2)	0.006 (2)
C21	0.020 (2)	0.028 (3)	0.036 (3)	0.000 (2)	0.006 (2)	0.004 (2)
C22	0.022 (3)	0.022 (3)	0.032 (3)	0.003 (2)	0.003 (2)	−0.004 (2)
C23	0.020 (2)	0.025 (3)	0.017 (2)	0.0019 (19)	0.0054 (18)	0.0005 (18)
C24	0.029 (3)	0.028 (3)	0.024 (2)	0.004 (2)	0.005 (2)	0.003 (2)
C25	0.025 (3)	0.032 (3)	0.026 (2)	0.007 (2)	0.007 (2)	−0.001 (2)
C26	0.031 (3)	0.041 (3)	0.023 (2)	−0.004 (2)	0.010 (2)	−0.003 (2)
C27	0.028 (3)	0.043 (3)	0.020 (2)	0.001 (2)	0.002 (2)	0.007 (2)
C28	0.022 (2)	0.029 (3)	0.029 (3)	0.005 (2)	0.008 (2)	0.001 (2)
C29	0.018 (2)	0.023 (3)	0.024 (2)	−0.0015 (19)	−0.0011 (18)	0.0039 (18)
C30	0.025 (3)	0.028 (3)	0.043 (3)	0.004 (2)	0.012 (2)	0.001 (2)
C31	0.032 (3)	0.028 (3)	0.069 (4)	0.010 (2)	0.011 (3)	−0.002 (3)
C32	0.039 (3)	0.030 (3)	0.062 (4)	0.006 (3)	0.004 (3)	0.016 (3)
C33	0.037 (3)	0.034 (3)	0.041 (3)	−0.004 (2)	0.008 (2)	0.013 (2)
C34	0.031 (3)	0.030 (3)	0.029 (3)	−0.002 (2)	0.005 (2)	0.004 (2)
C35	0.020 (2)	0.020 (2)	0.024 (2)	0.0007 (19)	0.0077 (19)	−0.0015 (18)
C36	0.028 (3)	0.041 (3)	0.025 (2)	−0.005 (2)	0.005 (2)	0.006 (2)
C37	0.021 (3)	0.056 (4)	0.039 (3)	−0.004 (2)	0.004 (2)	0.012 (3)
C38	0.033 (3)	0.028 (3)	0.046 (3)	0.002 (2)	0.020 (3)	0.005 (2)
C39	0.045 (3)	0.033 (3)	0.029 (3)	0.003 (2)	0.019 (2)	−0.004 (2)
C40	0.021 (3)	0.039 (3)	0.027 (2)	0.002 (2)	0.007 (2)	−0.007 (2)
Pd2	0.01819 (17)	0.01557 (18)	0.01483 (15)	−0.00133 (14)	0.00368 (13)	0.00039 (13)
I2	0.03742 (19)	0.02015 (17)	0.02770 (16)	−0.01020 (14)	0.00826 (14)	−0.00112 (12)
P3	0.0172 (6)	0.0191 (6)	0.0185 (5)	−0.0013 (5)	0.0018 (5)	0.0001 (4)
P4	0.0252 (6)	0.0162 (6)	0.0185 (6)	−0.0011 (5)	0.0028 (5)	0.0007 (4)
N3	0.030 (2)	0.019 (2)	0.025 (2)	0.0029 (17)	0.0068 (17)	0.0002 (16)
N4	0.034 (2)	0.029 (2)	0.032 (2)	−0.0095 (19)	0.0102 (19)	0.0030 (18)
C41	0.020 (2)	0.016 (2)	0.014 (2)	−0.0028 (18)	0.0014 (17)	0.0031 (16)
C42	0.046 (3)	0.015 (3)	0.028 (3)	0.000 (2)	0.006 (2)	0.0018 (19)
C43	0.040 (3)	0.024 (3)	0.032 (3)	−0.012 (2)	0.002 (2)	0.007 (2)
C44	0.028 (3)	0.025 (3)	0.020 (2)	−0.002 (2)	0.0084 (19)	0.0039 (19)
C45	0.022 (2)	0.018 (2)	0.018 (2)	0.0000 (19)	−0.0003 (18)	−0.0004 (17)
C46	0.027 (3)	0.024 (3)	0.039 (3)	0.003 (2)	0.009 (2)	0.006 (2)
C47	0.039 (3)	0.026 (3)	0.042 (3)	0.012 (2)	0.008 (3)	0.003 (2)
C48	0.041 (3)	0.022 (3)	0.033 (3)	−0.001 (2)	0.002 (2)	0.004 (2)
C49	0.041 (3)	0.034 (3)	0.026 (3)	−0.009 (2)	0.007 (2)	0.002 (2)
C50	0.031 (3)	0.026 (3)	0.017 (2)	−0.001 (2)	0.0050 (19)	0.0016 (18)
C51	0.020 (2)	0.017 (2)	0.018 (2)	0.0002 (18)	0.0029 (17)	−0.0016 (17)
C52	0.024 (3)	0.024 (3)	0.026 (2)	0.002 (2)	0.004 (2)	0.0033 (19)
C53	0.037 (3)	0.021 (3)	0.026 (2)	−0.003 (2)	0.012 (2)	−0.002 (2)
C54	0.029 (3)	0.030 (3)	0.020 (2)	−0.005 (2)	0.008 (2)	−0.0019 (19)
C55	0.025 (3)	0.033 (3)	0.021 (2)	−0.002 (2)	0.0024 (19)	0.007 (2)
C56	0.021 (2)	0.024 (3)	0.027 (2)	0.003 (2)	0.006 (2)	0.0034 (19)
C57	0.017 (2)	0.023 (3)	0.025 (2)	0.0007 (19)	0.0046 (19)	0.0010 (19)
C58	0.029 (3)	0.036 (3)	0.037 (3)	−0.003 (2)	0.013 (2)	−0.008 (2)
C59	0.043 (3)	0.035 (3)	0.040 (3)	0.002 (3)	0.024 (3)	−0.008 (2)
C60	0.024 (3)	0.036 (3)	0.049 (3)	0.003 (2)	0.017 (2)	0.011 (2)
C61	0.028 (3)	0.051 (4)	0.040 (3)	−0.013 (3)	0.007 (2)	0.003 (3)
C62	0.033 (3)	0.046 (3)	0.028 (3)	−0.012 (3)	0.007 (2)	−0.004 (2)
C63	0.042 (3)	0.019 (2)	0.017 (2)	−0.008 (2)	0.006 (2)	−0.0005 (18)
C64	0.068 (4)	0.044 (4)	0.025 (3)	−0.028 (3)	−0.002 (3)	0.009 (2)
C65	0.098 (6)	0.051 (4)	0.024 (3)	−0.046 (4)	−0.001 (3)	0.005 (3)
C66	0.123 (7)	0.049 (4)	0.034 (3)	−0.044 (4)	0.041 (4)	−0.018 (3)
C67	0.104 (6)	0.035 (4)	0.072 (5)	−0.011 (4)	0.066 (4)	−0.012 (3)
C68	0.068 (4)	0.026 (3)	0.043 (3)	−0.001 (3)	0.034 (3)	0.001 (2)
C69	0.027 (3)	0.022 (3)	0.019 (2)	−0.001 (2)	−0.0001 (19)	0.0010 (18)
C70	0.033 (3)	0.029 (3)	0.023 (2)	−0.003 (2)	0.003 (2)	−0.003 (2)
C71	0.047 (3)	0.029 (3)	0.031 (3)	−0.003 (3)	0.009 (2)	−0.011 (2)
C72	0.040 (3)	0.024 (3)	0.037 (3)	−0.010 (2)	−0.001 (2)	−0.004 (2)
C73	0.026 (3)	0.028 (3)	0.036 (3)	−0.002 (2)	0.003 (2)	0.004 (2)
C74	0.026 (3)	0.024 (3)	0.027 (2)	0.002 (2)	0.002 (2)	0.001 (2)
C75	0.022 (2)	0.015 (2)	0.038 (3)	−0.0052 (19)	0.001 (2)	0.001 (2)
C76	0.033 (3)	0.024 (3)	0.041 (3)	0.001 (2)	0.013 (2)	−0.002 (2)
C77	0.050 (4)	0.029 (3)	0.062 (4)	0.007 (3)	0.027 (3)	0.000 (3)
C78	0.038 (3)	0.022 (3)	0.098 (5)	0.007 (3)	0.029 (4)	0.013 (3)
C79	0.030 (3)	0.032 (4)	0.079 (5)	0.002 (3)	0.000 (3)	0.015 (3)
C80	0.036 (3)	0.020 (3)	0.056 (3)	−0.001 (2)	−0.003 (3)	0.005 (2)

### Geometric parameters (Å, º)

**Table:**

Pd1—C1	2.003 (4)	Pd2—C41	1.992 (4)
Pd1—P1	2.3373 (11)	Pd2—P4	2.3246 (12)
Pd1—P2	2.3383 (11)	Pd2—P3	2.3263 (11)
Pd1—I1	2.7025 (4)	Pd2—I2	2.6990 (4)
P1—C11	1.820 (4)	P3—C57	1.819 (4)
P1—C17	1.823 (4)	P3—C51	1.823 (4)
P1—C5	1.832 (4)	P3—C45	1.832 (4)
P2—C23	1.821 (4)	P4—C75	1.807 (5)
P2—C35	1.829 (4)	P4—C63	1.822 (5)
P2—C29	1.833 (4)	P4—C69	1.829 (4)
N1—C1	1.328 (5)	N3—C41	1.324 (5)
N1—C2	1.344 (6)	N3—C42	1.353 (6)
N2—C4	1.321 (5)	N4—C44	1.319 (5)
N2—C3	1.335 (6)	N4—C43	1.338 (6)
C1—C4	1.394 (5)	C41—C44	1.391 (6)
C2—C3	1.372 (6)	C42—C43	1.382 (7)
C2—H2	0.9500	C42—H42	0.9500
C3—H3	0.9500	C43—H43	0.9500
C4—H4	0.9500	C44—H44	0.9500
C5—C10	1.386 (6)	C45—C50	1.384 (6)
C5—C6	1.393 (6)	C45—C46	1.387 (6)
C6—C7	1.394 (6)	C46—C47	1.382 (6)
C6—H6	0.9500	C46—H46	0.9500
C7—C8	1.378 (7)	C47—C48	1.368 (7)
C7—H7	0.9500	C47—H47	0.9500
C8—C9	1.364 (7)	C48—C49	1.371 (7)
C8—H8	0.9500	C48—H48	0.9500
C9—C10	1.390 (6)	C49—C50	1.382 (6)
C9—H9	0.9500	C49—H49	0.9500
C10—H10	0.9500	C50—H50	0.9500
C11—C12	1.389 (6)	C51—C52	1.386 (6)
C11—C16	1.397 (6)	C51—C56	1.395 (6)
C12—C13	1.379 (6)	C52—C53	1.385 (6)
C12—H12	0.9500	C52—H52	0.9500
C13—C14	1.373 (6)	C53—C54	1.372 (6)
C13—H13	0.9500	C53—H53	0.9500
C14—C15	1.382 (7)	C54—C55	1.386 (6)
C14—H14	0.9500	C54—H54	0.9500
C15—C16	1.397 (6)	C55—C56	1.377 (6)
C15—H15	0.9500	C55—H55	0.9500
C16—H16	0.9500	C56—H56	0.9500
C17—C18	1.388 (6)	C57—C62	1.380 (6)
C17—C22	1.393 (6)	C57—C58	1.387 (6)
C18—C19	1.389 (6)	C58—C59	1.391 (6)
C18—H18	0.9500	C58—H58	0.9500
C19—C20	1.377 (7)	C59—C60	1.366 (7)
C19—H19	0.9500	C59—H59	0.9500
C20—C21	1.366 (6)	C60—C61	1.377 (7)
C20—H20	0.9500	C60—H60	0.9500
C21—C22	1.388 (6)	C61—C62	1.386 (6)
C21—H21	0.9500	C61—H61	0.9500
C22—H22	0.9500	C62—H62	0.9500
C23—C24	1.389 (6)	C63—C68	1.378 (7)
C23—C28	1.401 (6)	C63—C64	1.383 (6)
C24—C25	1.385 (6)	C64—C65	1.412 (8)
C24—H24	0.9500	C64—H64	0.9500
C25—C26	1.371 (6)	C65—C66	1.344 (9)
C25—H25	0.9500	C65—H65	0.9500
C26—C27	1.396 (6)	C66—C67	1.375 (9)
C26—H26	0.9500	C66—H66	0.9500
C27—C28	1.377 (6)	C67—C68	1.382 (7)
C27—H27	0.9500	C67—H67	0.9500
C28—H28	0.9500	C68—H68	0.9500
C29—C30	1.380 (6)	C69—C70	1.386 (6)
C29—C34	1.392 (6)	C69—C74	1.398 (6)
C30—C31	1.398 (7)	C70—C71	1.387 (6)
C30—H30	0.9500	C70—H70	0.9500
C31—C32	1.396 (7)	C71—C72	1.378 (7)
C31—H31	0.9500	C71—H71	0.9500
C32—C33	1.357 (7)	C72—C73	1.367 (7)
C32—H32	0.9500	C72—H72	0.9500
C33—C34	1.376 (6)	C73—C74	1.397 (6)
C33—H33	0.9500	C73—H73	0.9500
C34—H34	0.9500	C74—H74	0.9500
C35—C40	1.388 (6)	C75—C76	1.375 (6)
C35—C36	1.388 (6)	C75—C80	1.409 (6)
C36—C37	1.381 (6)	C76—C77	1.394 (6)
C36—H36	0.9500	C76—H76	0.9500
C37—C38	1.380 (7)	C77—C78	1.357 (8)
C37—H37	0.9500	C77—H77	0.9500
C38—C39	1.372 (7)	C78—C79	1.372 (8)
C38—H38	0.9500	C78—H78	0.9500
C39—C40	1.388 (6)	C79—C80	1.393 (7)
C39—H39	0.9500	C79—H79	0.9500
C40—H40	0.9500	C80—H80	0.9500
			
C1—Pd1—P1	87.86 (12)	C41—Pd2—P4	87.27 (12)
C1—Pd1—P2	87.21 (12)	C41—Pd2—P3	88.26 (12)
P1—Pd1—P2	173.12 (4)	P4—Pd2—P3	170.29 (4)
C1—Pd1—I1	179.38 (12)	C41—Pd2—I2	178.44 (12)
P1—Pd1—I1	92.46 (3)	P4—Pd2—I2	92.22 (3)
P2—Pd1—I1	92.42 (3)	P3—Pd2—I2	92.47 (3)
C11—P1—C17	105.01 (19)	C57—P3—C51	107.2 (2)
C11—P1—C5	102.72 (19)	C57—P3—C45	105.4 (2)
C17—P1—C5	102.8 (2)	C51—P3—C45	101.44 (19)
C11—P1—Pd1	111.78 (14)	C57—P3—Pd2	104.32 (14)
C17—P1—Pd1	117.08 (14)	C51—P3—Pd2	120.03 (14)
C5—P1—Pd1	115.82 (14)	C45—P3—Pd2	117.31 (14)
C23—P2—C35	108.0 (2)	C75—P4—C63	108.7 (2)
C23—P2—C29	100.2 (2)	C75—P4—C69	102.8 (2)
C35—P2—C29	103.6 (2)	C63—P4—C69	102.2 (2)
C23—P2—Pd1	118.23 (14)	C75—P4—Pd2	115.70 (16)
C35—P2—Pd1	108.57 (14)	C63—P4—Pd2	111.34 (16)
C29—P2—Pd1	116.91 (14)	C69—P4—Pd2	114.98 (14)
C1—N1—C2	116.7 (4)	C41—N3—C42	116.5 (4)
C4—N2—C3	114.9 (4)	C44—N4—C43	114.6 (4)
N1—C1—C4	119.8 (4)	N3—C41—C44	120.3 (4)
N1—C1—Pd1	119.9 (3)	N3—C41—Pd2	120.4 (3)
C4—C1—Pd1	120.3 (3)	C44—C41—Pd2	119.3 (3)
N1—C2—C3	122.1 (4)	N3—C42—C43	121.6 (4)
N1—C2—H2	119.0	N3—C42—H42	119.2
C3—C2—H2	119.0	C43—C42—H42	119.2
N2—C3—C2	122.3 (4)	N4—C43—C42	122.4 (4)
N2—C3—H3	118.9	N4—C43—H43	118.8
C2—C3—H3	118.9	C42—C43—H43	118.8
N2—C4—C1	124.3 (4)	N4—C44—C41	124.5 (4)
N2—C4—H4	117.8	N4—C44—H44	117.7
C1—C4—H4	117.8	C41—C44—H44	117.7
C10—C5—C6	119.3 (4)	C50—C45—C46	118.7 (4)
C10—C5—P1	122.4 (4)	C50—C45—P3	117.5 (3)
C6—C5—P1	117.9 (3)	C46—C45—P3	123.8 (3)
C5—C6—C7	119.8 (4)	C47—C46—C45	120.2 (5)
C5—C6—H6	120.1	C47—C46—H46	119.9
C7—C6—H6	120.1	C45—C46—H46	119.9
C8—C7—C6	120.4 (5)	C48—C47—C46	120.5 (5)
C8—C7—H7	119.8	C48—C47—H47	119.7
C6—C7—H7	119.8	C46—C47—H47	119.7
C9—C8—C7	119.6 (5)	C47—C48—C49	119.9 (5)
C9—C8—H8	120.2	C47—C48—H48	120.1
C7—C8—H8	120.2	C49—C48—H48	120.1
C8—C9—C10	121.1 (5)	C48—C49—C50	120.1 (5)
C8—C9—H9	119.5	C48—C49—H49	120.0
C10—C9—H9	119.5	C50—C49—H49	120.0
C5—C10—C9	119.8 (5)	C49—C50—C45	120.6 (4)
C5—C10—H10	120.1	C49—C50—H50	119.7
C9—C10—H10	120.1	C45—C50—H50	119.7
C12—C11—C16	119.3 (4)	C52—C51—C56	119.1 (4)
C12—C11—P1	118.1 (3)	C52—C51—P3	121.4 (3)
C16—C11—P1	122.5 (3)	C56—C51—P3	119.5 (3)
C13—C12—C11	120.3 (4)	C53—C52—C51	120.1 (4)
C13—C12—H12	119.9	C53—C52—H52	119.9
C11—C12—H12	119.9	C51—C52—H52	119.9
C14—C13—C12	120.7 (4)	C54—C53—C52	120.4 (4)
C14—C13—H13	119.7	C54—C53—H53	119.8
C12—C13—H13	119.7	C52—C53—H53	119.8
C13—C14—C15	120.0 (4)	C53—C54—C55	120.0 (4)
C13—C14—H14	120.0	C53—C54—H54	120.0
C15—C14—H14	120.0	C55—C54—H54	120.0
C14—C15—C16	120.0 (4)	C56—C55—C54	119.9 (4)
C14—C15—H15	120.0	C56—C55—H55	120.0
C16—C15—H15	120.0	C54—C55—H55	120.0
C15—C16—C11	119.7 (4)	C55—C56—C51	120.4 (4)
C15—C16—H16	120.2	C55—C56—H56	119.8
C11—C16—H16	120.2	C51—C56—H56	119.8
C18—C17—C22	118.6 (4)	C62—C57—C58	119.5 (4)
C18—C17—P1	120.3 (3)	C62—C57—P3	123.0 (4)
C22—C17—P1	121.1 (3)	C58—C57—P3	117.3 (4)
C17—C18—C19	120.0 (4)	C57—C58—C59	119.7 (5)
C17—C18—H18	120.0	C57—C58—H58	120.2
C19—C18—H18	120.0	C59—C58—H58	120.2
C20—C19—C18	120.5 (4)	C60—C59—C58	120.8 (5)
C20—C19—H19	119.8	C60—C59—H59	119.6
C18—C19—H19	119.8	C58—C59—H59	119.6
C21—C20—C19	120.2 (4)	C59—C60—C61	119.3 (5)
C21—C20—H20	119.9	C59—C60—H60	120.4
C19—C20—H20	119.9	C61—C60—H60	120.4
C20—C21—C22	119.9 (4)	C60—C61—C62	120.9 (5)
C20—C21—H21	120.1	C60—C61—H61	119.6
C22—C21—H21	120.1	C62—C61—H61	119.6
C21—C22—C17	120.9 (4)	C57—C62—C61	119.8 (5)
C21—C22—H22	119.6	C57—C62—H62	120.1
C17—C22—H22	119.6	C61—C62—H62	120.1
C24—C23—C28	118.5 (4)	C68—C63—C64	119.1 (5)
C24—C23—P2	121.0 (3)	C68—C63—P4	117.3 (4)
C28—C23—P2	120.3 (3)	C64—C63—P4	123.4 (4)
C25—C24—C23	120.0 (4)	C63—C64—C65	118.4 (6)
C25—C24—H24	120.0	C63—C64—H64	120.8
C23—C24—H24	120.0	C65—C64—H64	120.8
C26—C25—C24	121.3 (4)	C66—C65—C64	121.2 (6)
C26—C25—H25	119.3	C66—C65—H65	119.4
C24—C25—H25	119.3	C64—C65—H65	119.4
C25—C26—C27	119.4 (4)	C65—C66—C67	120.7 (6)
C25—C26—H26	120.3	C65—C66—H66	119.6
C27—C26—H26	120.3	C67—C66—H66	119.6
C28—C27—C26	119.6 (4)	C66—C67—C68	118.7 (6)
C28—C27—H27	120.2	C66—C67—H67	120.6
C26—C27—H27	120.2	C68—C67—H67	120.6
C27—C28—C23	121.2 (4)	C63—C68—C67	121.7 (6)
C27—C28—H28	119.4	C63—C68—H68	119.1
C23—C28—H28	119.4	C67—C68—H68	119.1
C30—C29—C34	119.1 (4)	C70—C69—C74	118.9 (4)
C30—C29—P2	121.9 (4)	C70—C69—P4	121.3 (4)
C34—C29—P2	119.0 (4)	C74—C69—P4	119.1 (3)
C29—C30—C31	120.2 (5)	C69—C70—C71	119.8 (5)
C29—C30—H30	119.9	C69—C70—H70	120.1
C31—C30—H30	119.9	C71—C70—H70	120.1
C32—C31—C30	119.0 (5)	C72—C71—C70	120.7 (5)
C32—C31—H31	120.5	C72—C71—H71	119.6
C30—C31—H31	120.5	C70—C71—H71	119.6
C33—C32—C31	120.8 (5)	C73—C72—C71	120.4 (5)
C33—C32—H32	119.6	C73—C72—H72	119.8
C31—C32—H32	119.6	C71—C72—H72	119.8
C32—C33—C34	120.0 (5)	C72—C73—C74	119.5 (5)
C32—C33—H33	120.0	C72—C73—H73	120.3
C34—C33—H33	120.0	C74—C73—H73	120.3
C33—C34—C29	120.8 (5)	C73—C74—C69	120.6 (4)
C33—C34—H34	119.6	C73—C74—H74	119.7
C29—C34—H34	119.6	C69—C74—H74	119.7
C40—C35—C36	118.6 (4)	C76—C75—C80	118.3 (5)
C40—C35—P2	117.5 (3)	C76—C75—P4	119.9 (4)
C36—C35—P2	123.8 (3)	C80—C75—P4	121.7 (4)
C37—C36—C35	120.3 (4)	C75—C76—C77	121.3 (5)
C37—C36—H36	119.8	C75—C76—H76	119.4
C35—C36—H36	119.8	C77—C76—H76	119.4
C38—C37—C36	120.8 (5)	C78—C77—C76	119.5 (5)
C38—C37—H37	119.6	C78—C77—H77	120.2
C36—C37—H37	119.6	C76—C77—H77	120.2
C39—C38—C37	119.3 (5)	C77—C78—C79	121.3 (5)
C39—C38—H38	120.3	C77—C78—H78	119.4
C37—C38—H38	120.3	C79—C78—H78	119.4
C38—C39—C40	120.4 (4)	C78—C79—C80	119.7 (5)
C38—C39—H39	119.8	C78—C79—H79	120.1
C40—C39—H39	119.8	C80—C79—H79	120.1
C35—C40—C39	120.5 (4)	C79—C80—C75	119.9 (5)
C35—C40—H40	119.7	C79—C80—H80	120.0
C39—C40—H40	119.7	C75—C80—H80	120.0

### Hydrogen-bond geometry (Å, º)

Cg1, Cg2, Cg3, Cg4 and Cg5 are the centroids of the C11–C16, C17–C22, C51–C56, C63–C68 and C75–C80 rings, respectively.

**Table:**

*D*—H···*A*	*D*—H	H···*A*	*D*···*A*	*D*—H···*A*
C2—H2···*Cg*5^i^	0.95	2.99	3.898 (5)	160
C7—H7···*Cg*3	0.95	3.00	3.930 (5)	168
C25—H25···*Cg*1^ii^	0.95	2.91	3.769 (5)	151
C42—H42···*Cg*2^ii^	0.95	3.00	3.944 (5)	176
C53—H53···*Cg*4^i^	0.95	2.80	3.617 (5)	145

Symmetry codes: (i) *x*, −*y*+3/2, *z*+1/2; (ii) *x*, −*y*+1/2, *z*−1/2.
